# The ocular surface after successful glaucoma filtration surgery: a clinical, in vivo confocal microscopy, and immune-cytology study

**DOI:** 10.1038/s41598-019-47823-z

**Published:** 2019-08-05

**Authors:** Luca Agnifili, Lorenza Brescia, Francesco Oddone, Matteo Sacchi, Erminia D’Ugo, Guido Di Marzio, Fabiana Perna, Ciro Costagliola, Rodolfo Mastropasqua

**Affiliations:** 1 Ophthalmology Clinic, Department of Medicine and Aging Science, University G, d’Annunzio of Chieti-Pescara, Chieti, Italy; 2 IRCCS Fondazione G.B. Bietti, Roma, Italy; 3 Ospedale San Giuseppe, Milano, Italy; 4 International Agency of Prevention of Blindness, Roma, Italy; 5 Department of Medicine and Health Sciences “V. Tiberio”, University of Molise, Campobasso, Italy; 6 Bristol Eye Hospital, University Hospitals NHS Foundation Trust, Bristol, BS1 2LX United Kingdom

**Keywords:** Conjunctival diseases, Diseases, Optic nerve diseases, Glaucoma, Corneal diseases

## Abstract

We investigated the ocular surface (OS) system modifications after completely successful glaucoma surgery in thirty-eight patients undergoing trabeculectomy (surgical group), using laser scanning confocal microscopy (LSCM) and impression cytology (IC). Twenty-six medically controlled glaucomatous patients served as controls (medical group). LSCM, IC, and the ocular surface disease index (OSDI) and National Eye Institute Visual Function Questionnaire-25 (NEI VFQ-25) questionnaires, were performed at baseline and after six months. The main outcomes were: goblet cell density (GCD), limbal dendritic cell density (LDCD), subbasal corneal nerve inhomogeneity (SCNI), Meibomian gland density and inhomogeneity (MGD, MGI), and HLA-DR positivity. There were no significant baseline differences between groups. At the sixth month, the surgical group showed a GCD increase (p < 0.001), and a LDCD, SCNI, MGI, HLA-DR (p < 0.001), OSDI and NEI VFQ-25 scores decrease (p < 0.05). The medical group did not show significant OS modifications, showing LSCM and IC parameters significantly worse compared to the surgical group (p < 0.001). The OSDI score correlated with GCD, MGI, SCNI, LDCD, and HLA-DR (p < 0.001; p < 0.05; p < 0.01). The present study found that the whole OS system objectively improved after completely successful glaucoma filtration surgery. These changes positively affected the OSDI score, but not the NEI VFQ-25 score.

## Introduction

The daily instillation of intra-ocular pressure (IOP) lowering drugs represents the proven therapeutic strategy to control the retinal ganglion cell loss in patients with glaucoma. However, a high number of patients requires multiple eye drops instillations during the day to control the disease^1^.

The literature widely demonstrated that the chronic use of anti-glaucoma medications negatively affects tissues composing the ocular surface (OS) system^2–9^. Cornea, corneo-scleral limbus, conjunctiva, and adnexa undergo crucial alterations that depends on the presence and cumulative daily dose of preservative and active compounds, the number of eye drops instilled per day, and the duration of therapy. The alterations of each OS component take part in the development of the iatrogenic ocular surface disease (OSD), which is a particular form of evaporative dry eye affecting a large part of the glaucoma population^10^. The presence of a symptomatic OSD represents a big challenge in the glaucoma management, because it reduces the adherence to therapy and worsens the patient quality of life (QoL)^11^. In addition, OS changes represent a recognized risk factor for the outcome of further glaucoma filtration surgery (GFS)^12–14^.

GFS is a consolidated approach in the management of glaucoma, with the potentiality to greatly reduce IOP^15^. When filtration surgery is completely successful it may potentially produce significant improvements of the OS system through different mechanisms. Consequently, the positive change of the OS status may produce potential benefits for the patient QoL; however, on the other hand, the presence of a filtering bleb may induce different OS alterations and maintain, at least in part, a mitigated form of OSD^16–18^. These aspects may probably account for the worse QoL experienced by surgically compared to medically treated glaucomatous patients^19^. To date, no previous study compared the *in vivo* and *ex vivo* features of the whole OS before and after completely successful filtration surgery. The aims of the present study were to compare before and six months after completely successful GFS: i) OS clinical tests, such as break-up-time (BUT), Schirmer test I (STI), and corneal fluorescein staining (CFS); ii) goblet cell density (GCD), limbal dendritic cell density (LDCD), subbasal corneal nerve inhomogeneity (SCNI), and Meibomian gland density and inhomogeneity (MGD, MGI) at laser scanning confocal microscopy (LSCM), and HLA-DR positivity at impression cytology (IC); iii) the ocular surface disease index (OSDI) score and 25-item National Eye Institute Visual Function Questionnaire (NEI VFQ) score.

## Results

At the sixth month, when topical steroids were tapered down, surgeries were completely successful in 28 patients (76.5%); six patients abandoned the study because required additional IOP lowering medications to control the disease. None of the considered patients received steroids before surgery or developed OSD during follow up, or used lubricants. No major intra- or post-operative complications occurred, and none of the patients underwent bleb needling procedures or anti-metabolite injections during the study period.

### Clinical data

The demographic and baseline clinical data of both groups are shown in Table 1. Baseline IOP was significantly higher in the surgical than in medical group, with a similar mean number of medications; after six months, the surgical group showed a 43% of IOP reduction, whereas did not significantly change in the medical group (Table 2).

**Table 1:** Demographics and clinical parameters of surgical and medical groups.

	Age (years ± SD)	Gender (M/F)	MD (dB ± SD)	Mean time on therapy (months ± SD)^§^	Last follow-up (months ± SD)
Surgical group	61.8 ± 9.5	16/12	−7.51 ± 1.81*	68.2 ± 4.2	6.8 ± 0.5
Medical group	59.8 ± 5.7	12/14	−6.21 ± 1.42	63.7 ± 6.1	6.6 ± 0.8

^*^P < 0.05 vs Medical Group.

^§^The time on therapy matched with the time of diagnosis.

**Table 2:** Baseline and 6-month clinical tests and questionnaire scores.

	Time point	IOP	Mean N° of medications	CFS	BUT	STI	OSDI	NEI VFQ-25
Surgical group	Baseline	29.1 ± 2.5^II^	3.01 ± 0.2^‡^	2.68 ± 1.31^‡^	8.00 ± 1.51^‡^	7.10 ± 1.80^‡^	55.40 ± 10.93^‡^	79.2 ± 15.1^‡^
	6^th^ month	16.7 ± 2.6	NA	1.70 ± 0.90^*†^	7.91 ± 1.88^‡^	6.92 ± 1.94^‡^	22.55 ± 13.07*^,†^	64.8 ± 9.3*^,†^
Medical group	Baseline	15.6 ± 2.3^§^	2.92 ± 0.3 ^§^	2.57 ± 0.38^§^	8.48 ± 1.49^§^	6.90 ± 1.20^§^	52.01 ± 0.80^§^	81.3 ± 4.8^§^
	6^th^ month	16.0 ± 0.7	2.91 ± 0.8	2.69 ± 0.61	7.99 ± 1.03	7.20 ± 1.90	49.82 ± 1.19	80.1 ± 5.1

CFS = corneal fluorescein staining (mean ± SD; Oxford scale).

BUT = break-up time (mean ± SD; sec).

STI = Schirmer test I (mean ± SD; mm).

OSDI = ocular surface disease index score (mean ± SD).

NEI VFQ-25 = National Eye Institute visual function questionnaire (mean ± SD).

IOP (mmHg) = intra-ocular pressure.

NA = not applicable.

^*^p < 0.05 vs baseline.

^†^p < 0.01 vs Medical group.

^‡^p = ns vs Medical group.

^§^p = ns vs 6^th^ month.

^II^p < 0.05 vs Medical group.

### OSDI questionnaire, ocular surface clinical tests, and NEI VFQ-25

The baseline OSDI questionnaire score, BUT, STI, corneal staining, and overall NEI VFQ-25 score were not significantly different between groups (Table 2). At six months after surgery, the OSDI questionnaire score reduced by 60%, from 55.40 to 22.55 (p < 0.001); conversely, the overall NEI VFQ-25 score reduced by 19%, from 79.2 to 64.8 (p > 0.01). While CFS significantly improved (p < 0.05), BUT and STI did not. After six months, no significant changes were found in the medical group, which showed CFS, OSDI significantly worse than the surgical group, with the NEI VFQ-25 score significantly better (p < 0.01).

### LSCM

**Figure 1:**
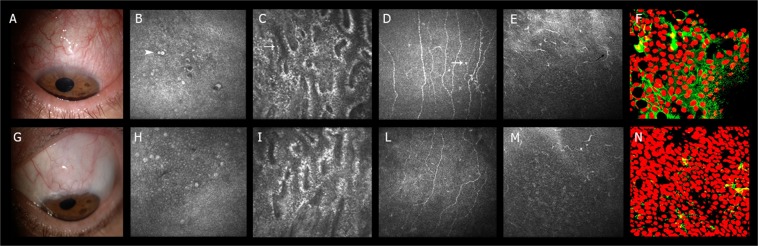
Ocular surface modifications after completely successful glaucoma filtration surgery. (**A–F**) Baseline assessment of the ocular surface and eyelids (**A**) in a patient candidate to filtration surgery. (**B**–**E**) indicate the confocal microscopy appearance of conjunctival GCs (arrowhead), MG and SCN patterns of inhomogeneity (hyper-reflective points, white arrow), and limbal DCs (black arrow), respectively. F shows the IC of the conjunctiva with the HLA-DR positivity represented in green. (**G–N**) Post-surgical assessment of the ocular surface and eyelids (**G**). At six months, GCs increased their density (**H**), whereas the MG and SCN patterns of inhomogeneity (**I**,**L**), the limbal DC density (**M**) and the conjunctival HLA-DR positivity (**N**), reduced.

Baseline and six-month LSCM parameters are reported in Table 3. GCs, limbal DCs, sub-basal corneal nerves, and MGs were easily recognized and were morphologically similar to those described in previous confocal studies^5–7,12,13^. At baseline, all confocal parameters were not significantly different between the surgical and medical groups. At the six-month after surgery, GCD increased (p < 0.001) while LDCD, MGI, and SCNI reduced (p < 0.01); conversely, no significant changes were found in the medical group. When comparing the 6^th^ month results between Groups, the surgical group showed significantly higher GCD (p < 0.001), and lower LDCD (p < 0.01), MGI and SCNI (p < 0.001) values with respect to the medical group. MGD did not show significant changes (Fig. 1B–E,H–M).

**Table 3:** Baseline and 6-month confocal and impression cytology parameters.

	Time point	GCD	LDCD	MGD	MGI	SCNI	HLA-DR
Surgical group	Baseline	33.12 ± 17.20^**^	64.71 ± 10.60^**^	157.24 ± 18.00^**,II^	2.29 ± 0.33	2.64 ± 0.31^**^	41.21 ± 7.15^**^
	6^th^ month	84.97 ± 38.58^*,‡^	35.85 ± 10.82^†,§^	150.57 ± 15.89	1.74 ± 0.28^†,‡^	1.71 ± 0.21^†‡^	22.34 ± 6.72^*,‡^
Medical group	Baseline	36.49 ± 11.37^II^	67.05 ± 12.79^II^	159.89 ± 12.50^II^	2.32 ± 0.29^II^	2.57 ± 0.34^II^	39.08 ± 0.83^II^
	6^th^ month	37.63 ± 6.38	68.90 ± 9.30	161.46 ± 9.17	2.28 ± 0.29	2.44 ± 0.18	41.99 ± 0.75

GCD = goblet cell density (mean ± SD; cells/mm^2^).

LDCD = limbal dendritic cell density (mean ± SD; cells/mm^2^).

MGD = Meibomian gland density (acinar units/mm^2^).

MGI = Meibomian gland inhomogeneity.

SCNI = Sub-basal corneal nerve inhomogeneity.

HLA-DR = %.

^*^p < 0.001 vs baseline.

^†^p < 0.01 vs baseline.

^‡^p < 0.001 vs Medical group (baseline and 6^th^ month).

^§^p < 0.01 vs Medical group (baseline and 6^th^ month).

^**^p = ns vs Medical group.

^II^p = ns vs 6^th^ month.

### Impression cytology

**Figure 2:**
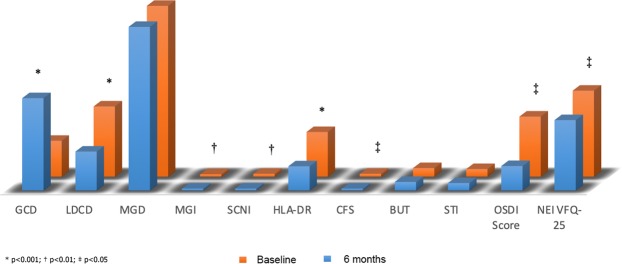
Bar graph representation of baseline (red bars) and 6 months (blue bars) clinical, confocal and impression cytology parameter changes in completely successful filtration surgery.

Table 3 shows baseline and six-month IC results; baseline HLA-DR positivity was not significantly different between groups. At six months HLA-DR significantly reduced from 41.21% to 22.34% (p < 0.001) (Fig. 1F,N) in the surgical group whereas did not change in the medical group; the six-month HLA-DR positivity was significantly lower in the surgical compared to the medical group (p < 0.001). Figure 2 summarizes six-months changes of LSCM, IC, and clinical parameters, and OSDI and NEI VFQ-25 scores compared to baseline.

### Correlations

Baseline and 6-month GCD negatively correlated with OSDI score (p < 0.01, r = −0.786 and p < 0.001, r = −0.819) and CFS (p < 0.05, r = −0.678 and p < 0.05, r = −0.613). Baseline and six-month MGI, SCNI, LDCD, and HLA-DR positively correlated with the OSDI score (p < 0.01, r = 0.699 and p < 0.01, r = 0.725; p < 0.01, r = 0.671 and p < 0.05, r = 0.744; p < 0.001, r = 0.856 and p < 0.05, r = 0.700; p < 0.001, r = 0.811 and p < 0.01, r = 0.789; respectively). Six-month HLA-DR positively correlated with LDCD, SCNI, and MGI (p < 0.01, r = 0.911; p < 0.01, r = 0.794; p < 0.05, r = 0.614), and negatively correlated with GCD (p < 0.01, r = −0.833).

## Discussion

Besides the IOP reduction, filtration surgery may produce additional beneficial effects for the eye with glaucoma, such as the improvement of the OS system. In our study, several factors contributed to the OS improvement, with the discontinuation of medical therapy and the use of post-operative steroids probably playing the most crucial roles.

The cessation of the toxic and immune stimulating effects of medications^4^ along with the therapeutic effects of steroids, suggest that the inflammation decrease may represent one of the patho-physiological way leading to the OS improvement.

However, the development of the filtration bleb, the use of intra-operative mitomycin-C, and the post-operative scarring processes within the conjunctiva may also concur to the final OS changes. Thus, the modified conditions of the OS system after completely successful GFS should be interpreted as a multifactorial response to surgery.

In more detail, inflammatory markers such as LDCD, SCN and MG inhomogeneity, and the HLA-DR expression presented a half reduction compared to pre-operative values. In addition, the CFS score reduction can be intended as a clinical proof of the inflammation decrease seen at LSCM and IC. On the other hand, the three-fold GCD increase along with the OSDI score reduction, indicated a potential improvement of dry eye.

Our work represents the first study proposing a multi-parametric analysis of the effects of surgery on the whole OS system and on the patient QoL. In fact, the evaluation of the global impact of GFS on OS did not previously arouse particular attention. Cvenkel and coworkers found that glaucoma surgery decreased the percentage of HLA-DR positive cells on the OS in the early post-operative period, because of the use of steroids^20^. However, they did not find further reduction of the HLA-DR positivity at longer follow-up. This discrepancy could be due to the fact that our patients had a lower mean baseline HLA-DR positivity (41% vs 56%), and a last follow-up slightly longer than that considered by Cvenkel *et al*. This could be relevant, since the HLA-DR expression tends to reduce six months after GFS^21^. The persistence of a mild sub-clinical level of inflammation also six months after completely successful surgery, can be due to the trans-conjunctival aqueous humor (AH) percolation, which carry AH derived inflammatory molecules on the OS^4,20^.

The decrease of the OS inflammation may have different positive effects, both for the bleb functionality and the overall OS status. In fact, a higher DC density within the ocular surface tissues was related to a higher risk of bleb dysfunction, and to worse signs and symptoms of OSD^4–7,13^. On the other hand, the less inflamed eyelid status may also contribute to improve the OS and maintain a good bleb function^22^.

When analyzing eyelids, though we found a reduction of MGI, we did not observe significant changes in MGD. This is in contrast with the study of Sagara and coworkers, who documented MG loss after MMC augmented trabeculectomy^18^. This could be due to the fact that they considered patients with a longer follow-up and evaluated the upper eyelid, where MGs may be more disturbed by the direct contact with the bleb.

Interesting changes affected also the subbasal nerve plexus. This structure represents an important site of analysis in presence of dry eye, showing a lower density and length of nerve fibers and branches, tortuosity, and reduced nerve fiber width^23^. Of note, these changes have been reported to be in line with the DC increase^24^.

However, to date, no direct confocal indicators of corneal nerve inflammation were investigated. In this study, we proposed for the first time a new potential indicator of subbasal nerve plexus inflammation, which is the SCNI. We observed that this parameter tend to reduce after completely successful GFS, indicating a mitigation of the OSD condition. In fact, the reduction of the hyper-reflective points between nerve fibers suggests a less local immune stimulation. The cessation of toxic triggers produced by the therapy discontinuation along with the use of post-operative steroids may be considered as the main determinants of the decreased inflammatory status of the subbasal nerve plexus. Nevertheless, further investigation should confirm whether SCNI could be considered an accurate marker of subbasal corneal alterations in presence of OSD.

The post-surgical GCD increase represents a crucial OS modification since GCs are considered as cytological markers of a good ocular surface status^25^. In fact, one of the most important indicators of OSD is represented by the GC loss; this aspect was also widely documented in patients with glaucoma-related OSD^4–7^. In our study, the post-operative GCD increase, along with the OSDI score reduction, can be intended as indicators of a potential dry eye improvement. As for the inflammatory markers, the medical therapy discontinuation and the use of steroids probably represent the main determinants of dry eye mitigation. The unmodified post-surgical BUT and STI values are not in agreement with these changes, even though one may suppose that cytological modifications may precede the clinical changes.

The post-surgical GCD increase is also crucial for a good bleb function, since GCs have been found to play a main role in the AH percolation through the bleb-wall epithelium. In fact, Amar and coworkers hypothesized that the transcellular pathway of the AH occur at the level of GCs toward the ocular surface^26^. This hypothesis was further supported by prospective studies that found that higher pre-surgical GCD values strongly correlated with the filtration surgery outcome^12,13^.

It is difficult to state which of the observed modifications have the greater impact on the better OS status. Nonetheless, given the significant correlations among all inflammatory parameters and OSDI score, one may hypothesize that the inflammation decrease may play a pivotal role.

Nevertheless, as stated above, other factors must be considered when interpreting the post-surgical OS system changes. (i) The mechanical effects of the new-formed filtration bleb may disturb the tear film stability, and induce a persistency of a significant form of dry eye^16,17,27,28^. (ii) The use of intra-operative MMC induces toxic effects on the OS system, by inhibiting the proliferation of corneal and conjunctival cells, and disturbing MGs^18,29^. This may lead to a worsening of all tear film components after surgery, thus limiting in part the final improvement of the OS. Nevertheless, the effects of MMC have been demonstrated to completely disappear six months after surgery, with goblet and epithelial cells recovering their normal density^30,31^. (iii) The use of post-operative steroids may have had a huge effect on the final OS modifications, because of the inflammation decrease and the inhibitory effects on the recruitment of sub-conjunctival fibroblasts in the inflammatory phase of the bleb formation. Thus, steroids affected, at least in part, the morphological characteristics of the bleb and its impact on the post-operative OS status^32^.

However, the choice to schedule the follow-up at the sixth month, when the effects of bleb manipulation, steroids, and early scarring processes are terminated, can reduce the impact of these factors on final results^30–33^. On the other hand, the high inter-patient variability in the duration of post-operative OS modifications does not allow to definitely confirm our findings. Certainly, an adjunctive follow-up would have added crucial information on the trend of modifications of the OS system.

The objective better OS conditions were only partially in line with the subjective improvement reported by patients: in fact, while the OSDI score improved, the NEI VFQ-25 score worsened. This is in accordance with previous evidences, such as those reported by the collaborative initial glaucoma treatment study (CIGTS)^34^. Even though this trial used different questionnaires to assess the QoL, a significant higher impact of surgery over medications on OSD symptoms was observed. This finding was reported to depend on the bleb formation which, especially when elevated and exposed, induced OS dysesthesia^11,17,27,28,35^. More recently, Guedes and coworkers reported that GFS is associated with a lower NEI VFQ-25 score compared to medications, especially in patients with an early glaucoma^36^. This seems in accordance with our study, that considered early to moderate glaucoma. In a different study, Ji and coworkers reported that 40% of patients with functioning filtering blebs suffered from symptomatic dry eye, with higher CFS scores and lower BUT values^16^. Interestingly, the occurrence of dry eye significantly correlated with the bleb features, being higher in patients with elevated and microcystic blebs. While we did not investigate the bleb morphology, we may hypothesize that the persistence of a mitigated form of dry eye along with the bleb formation could explain the worse NEI VFQ-25 score also in our cases. Thus, our results are in line with literature findings on the better QoL in the medically treated patients.

This study presents some limitations. First, we cannot ascertain whether the positive changes observed in the OS status is a primary effect of drug discontinuation, a secondary effect due to the use of topical steroids, or a mixed effect. Similarly, we cannot clearly state whether changes we observed would be the same even without the use of post-operative steroids. Second, we cannot state what is the best parameter expressing the OS improvement after surgery among those analyzed. Third, besides SCNI, we did not analyze structural corneal nerve parameters, such as fiber density, length and tortuosity, which have been found reduced in OSD^23,24^. Further studies are required to evaluate whether the positive effects of surgery seen on inflammatory corneal markers may positively affect also structural parameters. Fourth, the use of MMC could have potentially introduced significant biases in the final results, because of the cytotoxic effect of this agent. A control group receiving a MMC-independent glaucoma surgery would have permitted to clarify the impact of antimetabolites on OS changes. Finally, further studies with an adjunctive follow-up after the sixth month, will provide additional information on the OS changes and confirm or modify our initial results.

In closing, our study indicates that filtration surgery has a positive effect on the whole OS system; this is even more evident when comparing the OS features after surgery with patients under medical therapy. Nonetheless, because of the new formed filtration bleb, the objective OS improvement seems not to produce a manifestly positive effect on the patient QoL.

## Methods

### Patient selection

This was a 6-month, prospective, case-control study. The patients were treated in accordance with the criteria of the Declaration of Helsinki. Our institutional review board (Department of Medicine and Aging Science, G. d’Annunzio University of Chieti-Pescara, Chieti, Italy) evaluated and approved the project.

We consecutively enrolled twenty-eight Caucasian patients (28 eyes) with uncontrolled primary open angle glaucoma (POAG) scheduled to undergo mitomycin C (MMC) augmented filtration surgery (Surgical group). Twenty-six consecutive age and gender matched medically controlled glaucomatous subjects were used as controls (Medical group). Written informed consent was obtained from all subjects prior to enrolment.

Surgical group had to respect the followings inclusion criteria: (i) diagnosis of POAG with uncontrolled IOP (>21 mmHg, mean of three measurements) under maximal tolerated medical therapy (defined as the use of all tolerated classes of IOP lowering eye-drops including oral acetazolamide, without significant side effects and worsening of the QoL); (ii) therapy had to be unmodified during the last two months; (iii) significant progression of the optic nerve damage confirmed on three consecutive reliable visual fields (VF) ((Humphrey field analyzer II 750 (Carl Zeiss Meditec Inc., Dublin, CA) (30-2 test, full-threshold)). VF damage progression was assessed with the trend-based analysis of the HFA Guided Progression Analysis software: when the magnitude of visual field index (VFI) slope was worse than 1% per year with a P-value ≤ 0.05, the progression was considered clinically significant. If both eyes were eligible to surgery, the eye with the more advanced perimetric damage (glaucoma staging system 2 (GSS2))^37^, or the higher IOP or was included in the study.

The exclusion criteria were: history of previous GFS or ciliary body ablation, penetrating keratoplasty, retinal detachment; ocular diseases other than glaucoma, systemic or topical therapies in the last 6 months that could have modified the OS status, contact lens wear, pregnancy; laser trabeculoplasty or cataract surgery performed 6 months prior to enrollment, and cataract extraction planned at the time of filtering surgery or within six months thereafter.

The development of any form of OSD, the use of any type of eye-drops (including lubricants), the need to reintroduce anti-glaucoma medications or systemic drugs potentially affecting the OS during the follow up, were also considered exclusion criteria.

Filtration surgery was considered successful when at least 30% reduction from preoperative IOP was obtained at the sixth month; eyes that met the above criterion and were not on supplemental anti-glaucoma medical therapy were defined as complete success and were considered eligible for the study. In case of the need to use IOP lowering medications or to schedule needling procedures before the 6^th^ month, patients abandoned the study and received the appropriate therapy.

As per protocol, patients received unpreserved topical steroids eyedrops (dexamethasone 0.15%) for 12 weeks, tapered as follow: five times a day for 2 weeks, four times a day for 2 weeks, three times a day for 2 weeks, two times a day for 2 weeks, and one times a day for the following 4 weeks. Topical antibiotics were used for 2 weeks after trabeculectomy (unpreserved levofloxacin 5 mg/mL 4-fold daily).

Medical group had to respect the following inclusion criteria: BCVA greater than or equal to 8/10, a diagnosis of POAG, mean IOP controlled on maximal tolerated medical therapy, lower than 18 mm Hg and unmodified during the last two months, central corneal thickness (CCT) ranging from 530 to 570 µm, a glaucomatous appearance of the optic disc with consistent VF defects. Both eyes were evaluated, but one eye per control was randomly chosen (using a computer-generated random number list) for the statistical analysis.

Surgical groups underwent a weekly follow-up in the three months after surgery, and monthly afterwards; baseline and 6-month data were considered for the statistical analysis.

To limit the effects of intra-operative conjunctival manipulation, topical steroids, antibiotics, and MMC on the investigated parameters, the follow-up was scheduled six months after surgery.

### OSDI questionnaire, ocular surface clinical tests, NEI VFQ-25

After completion of the clinical assessment, a masked ophthalmologist (EDU) administered the OSDI and NEI VFQ-25 questionnaires to all participants. Afterwards, according to Dry Eye Work Shop (DEWS) guidelines, BUT, CFS, and STI (STI; 30 minutes after BUT measurements) were consecutively performed^38^. BUT was recorded as the average of three consecutive measurements; STI results were expressed as the length of the strip that was wet after 5 minutes; CFS was evaluated with 1% sodium fluorescein and scored according to the Oxford grading scale^39^. Clinical tests and questionnaires were administered at baseline and 6^th^ month.

### LSCM of the OS

LSCM (HRT III Rostock Cornea Module, Heidelberg Engineering, Heidelberg, Germany) was performed twenty-four hours after the clinical evaluation, in order to avoid misinterpretation due to the execution of tear film function tests, as previously described^5–7,12,40^. LSCM was performed to evaluate GCD, MGD and MGI, LDCD, and SCNI. For the examination of GCs, limbal DCs, sub-basal corneal nerves, and Meibomian glands we adopted the definitions and reference images consistently with those reported in previous confocal studies^5–7,12,40^.

### GC examination

GCs were examined throughout the epithelium of the lower bulbar conjunctiva, in order to avoid potential MMC-induced biases in the post-operative assessment. Forty images were acquired for each eye, and twelve high quality images without motion blur or compression lines were selected from the inferior nasal, central, and inferior temporal sectors (4 images for sector) to calculate the number of GCs (using the Cell Count Software of the device, in manual mode). GCs had to show the following features: an oval-shaped aspect with central or peripherally displaced nuclei, hyper-reflective and two/three times larger than the surrounding epithelial cells, dispersed or crowded in groups within the epithelium (15–30 µm of depth).

### MG examination

MGs were examined at the lower eyelid. After a partial eversion of the eyelid, MGs were analyzed along the entire lid length with minute horizontal movements, progressively moving from the most superficial to the deepest tissue that could be visualized with a satisfactory resolution. MGs had to appear as hyper-reflective acinar clusters connected by various ductules to a main duct, and the main duct to an external orifice. The MG examination was aimed at studying the acinar portion of the glandular complex, considering the following parameters: i) mean MGD (density of the acinar units for each 400 × 400 μm frame, manually marked and automatically calculated using the Cell Count software); ii) MGI of the glandular interstice and acinar wall; the inhomogeneity pattern represents a confocal indicator of inflammation, and is characterized by the evidence of hyper-reflective punctate elements dispersed within the tissue^5,41^. An arbitrary grading scale of 1–4 was adopted to quantify the inhomogeneity of both interstice and acinar wall, as previously described^5,41^. Ten images for each lid sector (nasal, central, and temporal) were acquired at every 10 μm of depth for a maximum depth of 50 μm; fifteen high quality MG images were considered to calculate the MGD and MGI.

### Limbal DCs examination

DCs were evaluated at the limbus avoiding the bleb site (3, 6, and 9 o’clock positions), after GC and MG assessment. DCs are normally located within the epithelium, the basal lamina, and the subbasal nerve plexus layer, at a depth ranging from 40 to 60 µm, and may present a mature or immature phenotype^6,7^. DCs were examined to determine their density (cells/mm^2^) using the instrument software, by averaging the numbers of cells from selected images, which were counted manually within a region of interest of standardized dimensions (250 × 250 µm). Forty-five images were acquired from each eye and at least 15 randomly selected high-quality images (5 for each limbal sector) were analyzed to determine the LDCD.

### Subbasal corneal nerve examination

The SCN examination was performed at the end of the confocal session, at the center of the cornea (2 × 2 mm), immediately beneath the basal epithelium and anterior to the Bowman’s membrane, at a depth of 50–80 µm. We evaluated the pattern of inhomogeneity of SCN in the same way proposed for MGs, were the evidence of multiple hyper-reflective punctate elements within the acinar wall and interstice were interpreted as inflammatory processes of MGs, in presence of dry eye^41^. An arbitrary grading scale 1–4 was adopted, with higher values indicating higher number of punctate reflective elements. In the same way, the SCNI corresponds to the evidence of hyper-reflective punctate elements dispersed between nerve fibers, and can be intended as a confocal feature of subbasal corneal nerve plexus inflammation. Thirty images were acquired from the central cornea, and ten high-quality images were analyzed to determine SNCI.

A single operator (LB) performed all confocal examinations and selected the images, which were evaluated by a second operator (MS). LB and BS were masked for the subject history and for grouping. Confocal session lasted less 5 to 10 minutes, and none of the patients reported complications related to the procedure.

As per protocol after filtration surgery, patients were treated with topical unpreserved dexamethasone 0.15% tapered in 12 weeks (four times a day for 4 weeks, three times a day for 4 weeks, and two times a day for the remaining 4 weeks), and with topical unpreserved levofloxacin 5 mg/mL (four times daily for 2 weeks).

LSCM was repeated six months after surgery, when therapy was completely terminated.

### IC of the conjunctiva

IC was performed 48 hours after LSCM to avoid any potential bias induced by the mechanical pressure, at the superior-temporal bulbar conjunctiva, with the eye in the opposite gaze, to evaluate the expression of the HLA-DR as previously reported^6^. IC was repeated at the 6-month follow-up at the superior-temporal sector, carefully avoiding any inadvertent bleb traumatism.

Five different fields for each IC sample were evaluated; positive (red nucleus and green cytoplasm) and negative (red nucleus) cells were counted. Two independent observers (GDM and FO) masked for the staining technique performed evaluations of specimens; digital images of representative areas were taken. At the end of the procedure, none of the patients reported complications related to the IC sampling.

The primary outcomes of the study were GCD, MGD and MGI, LDCD, and SCNI at LSCM, and HLAD-DR positivity at IC; the main secondary outcomes were the OSDI score, overall NEI VFQ-25. Correlations between baseline and 6-month confocal parameters and HLA-DR with baseline and 6-month OSDI, and NEI VFQ-25 score were evaluated; correlations between OSDI and NEI VFQ-25 scores at baseline and 6-month were also evaluated.

### Statistical analysis

Analysis was performed by SPSS Advanced Statistical TM 20.0 Software (2011; SPSS, Inc., Chicago, IL, USA). Student’s t-test and χ2 test were used to evaluate age and sex differences between groups. A 1-way ANOVA with post hoc Tukey for multi-comparison was used to analyze differences among groups. ANOVA repeated measures, was used to assess differences between baseline and 6 months data. Correlations among the confocal and IC variables and OSDI and NEI VFQ-25 scores were determined using a nonparametric measure by the Spearman’s index. A P value < 0.05 was considered statistically significant.

## Data Availability

We confirm that we have full access to all the data in the study and take responsibility for the integrity of the data and the accuracy of data analysis; data will be available upon request.
